# IRE1/Xbp1 promotes the clearance of poly(GR) dipeptide repeats in amyotrophic lateral sclerosis

**DOI:** 10.1016/j.jbc.2025.110764

**Published:** 2025-09-24

**Authors:** Yu Li, Dongyue Liu, Shuangxi Li

**Affiliations:** Shandong Provincial Key Laboratory of Animal Cell and Developmental Biology, School of Life Sciences, Shandong University, Qingdao, China

**Keywords:** *Drosophila melanogaster*, poly(GR), IRE1, Xbp1

## Abstract

Amyotrophic lateral sclerosis (ALS) and frontotemporal dementia (FTD) are neurodegenerative disorders characterized by the expansion of *GGGGCC (G4C2)* repeats in the *C9orf72* gene and progressive motor neuron degeneration. A key pathological hallmark of these diseases is the accumulation and cytoplasmic mislocalization of dipeptide repeat (DPR) proteins, particularly poly(GR), which are neurotoxic. Enhancing the clearance of poly(GR) represents a promising therapeutic strategy; however, the molecular mechanisms regulating poly(GR) turnover are not fully understood. Our previous work demonstrated that translationally stalled poly(GR) is targeted by the ribosome-associated quality control (RQC) pathway. In the present study, we identify the IRE1/Xbp1s signaling axis as an essential regulator of poly(GR) degradation. Ectopic expression of IRE1 or its downstream effector Xbp1s, as well as pharmacological activation of IRE1 using IXA4, significantly reduces poly(GR) protein levels in a *Drosophila* disease model, mammalian cell lines, fibroblasts derived from patients with C9orf72-ALS, and a C9orf72 transgenic mouse model. Mechanistically, RNA-sequencing analysis reveals that IRE1/Xbp1s signaling upregulates heat shock protein Hsp70Ba, which plays a critical role in maintaining poly(GR) proteostasis. Additionally, we show that the Rictor/AKT/VCP pathway contributes to the translational regulation and turnover of poly(GR). Importantly, activation of IRE1, either through ectopic expression or IXA4 treatment, mitigates motor neuron loss in the C9orf72 mouse model. Collectively, our findings highlight the IRE1/Xbp1s axis as a key modulator of poly(GR) clearance and suggest its therapeutic potential in ALS/FTD.

Hexanucleotide repeat expansions (*GGGGCC*) in the *C9orf72* gene represent the most prevalent genetic cause of amyotrophic lateral sclerosis (ALS) and frontotemporal dementia (FTD) (1, 2, 3, 4). A hallmark pathological feature of C9ORF72-associated ALS/FTD is the aberrant accumulation of dipeptide repeat proteins (DPRs), particularly poly(glycine-arginine) [poly(GR)], which are generated through repeat-associated non-AUG (RAN) translation of the expanded *G4C2* repeats (5, 6, 7). Poly(GR) is highly aggregation-prone and accumulates in both the cytoplasm and mitochondria, where it disrupts key cellular processes, including nucleocytoplasmic transport, mitochondrial function, and protein quality control (8, 9, 10). These disruptions lead to elevated cellular stress, ultimately contributing to neuronal dysfunction and degeneration (11, 12, 13). Mounting evidence indicates that poly(GR) accumulation is a major driver of toxicity in ALS, underscoring the urgent need to elucidate its regulatory mechanisms and identify potential therapeutic targets (14).

Poly(GR) proteostasis is governed by a delicate balance between its synthesis and degradation (15, 16). Its repetitive and aggregation-prone nature renders it particularly susceptible to aberrant accumulation, posing a significant challenge to cellular quality control systems (17, 18). Recent studies have highlighted the role of ribosome-associated quality control (RQC) in managing proteostasis and cellular stress burden during translation (19, 20, 21, 22). Translational stalling induced by poly(GR) sequences activates the RQC pathway (21, 23), facilitating the disassembly of stalled ribosomes and targeting of aberrant nascent peptides for degradation (24). Impairment of this process exacerbates poly(GR) accumulation and promotes proteotoxic stress (25), further linking defective translational surveillance to ALS pathogenesis.

The endoplasmic reticulum (ER) plays a central role in protein folding and quality control, with its function maintained by the unfolded protein response (UPR) (26, 27, 28, 29). Among the three canonical UPR sensors, inositol-requiring enzyme 1 (IRE1) is the most evolutionarily conserved and functions as a key regulator of ER proteostasis (30, 31). In response to ER stress, IRE1 undergoes oligomerization and autophosphorylation, initiating the unconventional splicing of X-box binding protein 1 (XBP1) mRNA. The resulting spliced isoform, XBP1s (32), acts as a transcription factor that induces a broad transcriptional program encompassing genes involved in protein folding, ER-associated degradation (ERAD), and lipid metabolism (33, 34). This adaptive response promotes cellular survival under proteotoxic conditions. Dysregulation of the IRE1-Xbp1 pathway has been implicated in numerous diseases, including neurodegeneration, cancer, and metabolic disorders (35, 36, 37, 38).

In this study, we identify a previously unrecognized role for the IRE1/Xbp1 signaling axis in regulating the proteostasis of poly(GR) independent of its canonical function in ER stress response. We demonstrate that activation of IRE1/Xbp1 significantly reduces poly(GR) protein accumulation and ameliorates associated proteotoxic phenotypes. These findings establish a novel, non-canonical function of the IRE1/Xbp1 branch in modulating pathogenic DPR burden and highlight its potential as a therapeutic target for ALS and FTD.

## Results

### The IRE1/Xbp1 axis attenuates poly(GR) expression and associated proteotoxicity

**Figure 1:**
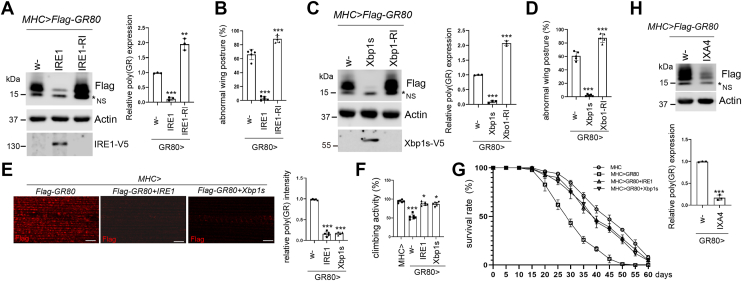
**Activation of the IRE1/Xbp1s axis facilitates poly(GR) clearance in a *Drosophila* disease model.***A*, Western blot analysis showing the effects of IRE1 overexpression or IRE1-RNAi on Flag-(GR)80 protein levels in *Drosophila* muscle tissue. NS stands for non-specific. The bar graph quantifies relative (GR)80 levels. Data points are presented as the mean ± S.D. ∗∗∗*p* < 0.001, ∗∗*p* < 0.01. Genotypes are: *+; MHC>Flag-GR80. IRE1-V5; MHC>Flag-GR80. +; MHC>Flag-GR80/IRE1-RI*. *B*, quantification of abnormal wing posture phenotypes induced by poly(GR) expression, with or without IRE1 overexpression or knockdown. Data points are presented as the mean ± S.D. ∗∗∗*p* < 0.001. *C*, Western blot analysis assessing the impact of Xbp1s overexpression or Xbp1-RNAi on Flag-(GR)80 levels in *Drosophila* muscle. Bar graph presents quantification of relative (GR)80 abundance. Data points are presented as the mean ± S.D. ∗∗∗*p* < 0.001. Genotypes are: *+; MHC>Flag-GR80. Xbp1s-V5; MHC>Flag-GR80. Xbp1-RI; MHC>Flag-GR80*. *D*, quantification of abnormal wing posture phenotype in flies co-expressing Flag-(GR)80 with either Xbp1s overexpression or Xbp1-RNAi. Data points are presented as the mean ± S.D.∗∗∗*p* < 0.001. *E*, representative immunofluorescence images of Flag-tagged poly(GR) in adult *Drosophila* muscle upon IRE1 or Xbp1s overexpression. Scale bar: 10 μm. The corresponding bar graph shows relative Flag-poly(GR) fluorescence intensity. Data points are presented as the mean ± S.D. ∗∗∗*p* < 0.001. Genotypes are: *+; MHC>Flag-GR80. IRE1-V5; MHC>Flag-GR80. Xbp1s-V5; MHC>Flag-GR80*. *F*, quantification of climbing ability in flies expressing Flag-(GR)80 alone or in combination with IRE1 or Xbp1s. Data points are presented as the mean ± S.D. ∗∗∗*p* < 0.001, ∗*p* < 0.05. *G*, survival analysis of flies expressing Flag-(GR)80 alone or in combination with IRE1 or Xbp1s, compared to control flies. Genotypes are: *+;MHC. +;MHC>Flag-GR80. IRE1-V5; MHC>Flag-GR80. Xbp1s-V5; MHC>Flag-GR80*. *H*, Western blot analysis of Flag-(GR)80 protein levels in *Drosophila* muscle tissue following administration of IXA4. The bar graph displays relative poly(GR) levels. Data points are presented as the mean ± S.D. ∗∗∗*p* < 0.001.

The Gal4/UAS system is a widely used genetic tool for driving targeted gene overexpression or knockdown in *Drosophila* (39). In our model, *MHC-Gal4>UAS-Flag-GR80*, poly(GR) dipeptide repeat proteins comprising 80 glycine-arginine residues (GR80) tagged with a Flag epitope were specifically expressed in muscle tissue under the control of the muscle-specific *MHC-Gal4* driver. Expression of GR80 in muscle led to pronounced neurotoxic phenotypes, notably an abnormal wing posture, which serves as a sensitive and quantifiable indicator of motor dysfunction (40). This phenotype mirrors the neuromuscular impairments characteristic of amyotrophic lateral sclerosis (ALS), making the *Drosophila* system a powerful *in vivo* model for investigating poly(GR)-induced pathogenesis and identifying potential therapeutic targets (41). Through a genetic modifier screen, we identified the IRE1/Xbp1 signaling axis as a key regulator of poly(GR) protein levels and toxicity. Ectopic expression of IRE1 significantly reduced poly(GR) protein accumulation and ameliorated the wing posture phenotype, whereas RNAi-mediated knockdown of IRE1 led to elevated poly(GR) levels and worsened the phenotype (Fig. 1, *A* and *B*). Similarly, overexpression of Xbp1s markedly suppressed poly(GR) expression and mitigated locomotor dysfunction, while loss of Xbp1 resulted in increased poly(GR) levels and enhanced wing defects (Fig. 1, *C* and *D*). These findings were further corroborated by immunostaining of muscle tissues, confirming reduced poly(GR) levels upon IRE1 or Xbp1s overexpression (Fig. 1*E*). Functionally, activation of the IRE1/Xbp1 pathway improved locomotor performance (Fig. 1*F*) and rescued the survival deficits associated with poly(GR) toxicity (Fig. 1*G*), indicating a protective role against poly(GR)-mediated cellular dysfunction. Furthermore, pharmacological activation of IRE1 through intrathoracic injection of IXA4 (42, 43) significantly suppressed poly(GR) protein expression (Fig. 1*H*), supporting the therapeutic potential of targeting this pathway. To determine whether poly(GR) overexpression activates the IRE1/Xbp1 pathway, we examined levels of IRE1, phosphorylated IRE1 and phosphorylated JNK upon poly(GR) overexpression. No significant changes were observed in the levels of p-IRE1 or p-JNK upon poly(GR) overexpression (Fig. S1). These results establish the IRE1/Xbp1 axis as a potent modulator of poly(GR) proteostasis and a promising target for mitigating dipeptide repeat protein-associated toxicity in ALS.

### Ectopic expression of IRE1 or Xbp1s promotes poly(GR) clearance in mammalian cells and ALS patient-derived fibroblasts

**Figure 2:**
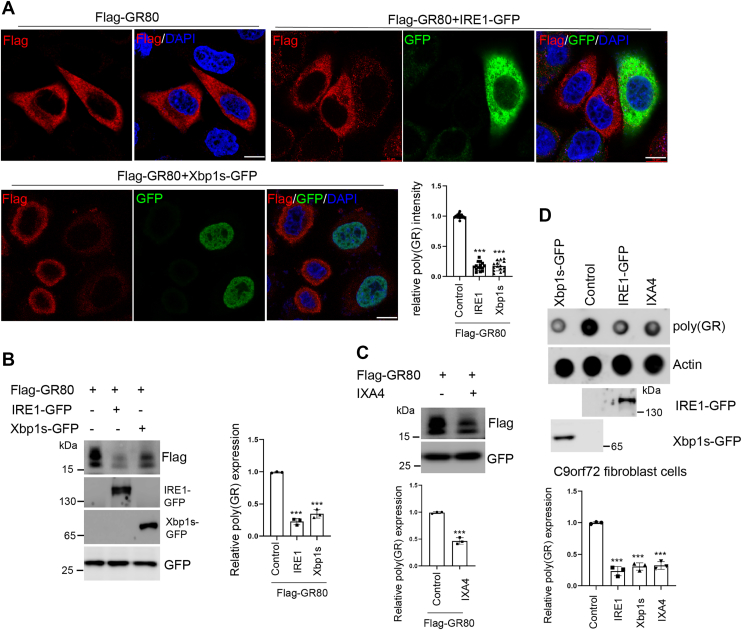
**The IRE1/Xbp1s pathway supresses poly(GR) protein levels in mammalian cells and C9orf72 patient-derived fibroblasts.***A*, representative immunofluorescence images of Flag-tagged (GR)80 in HeLa cells following overexpression of IRE1-GFP or Xbp1s-GFP. Scale bar: 10 μm. The adjacent bar graph quantifies relative Flag-(GR)80 fluorescence intensity. Data points are presented as the mean ± S.D. ∗∗∗*p* < 0.001. *B*, Western blot analysis depicting the impact of IRE1 or Xbp1s overexpression on Flag-(GR)80 protein levels in HEK293T cells. Quantification of relative (GR)80 abundance is shown in the accompanying bar graph. Data points are presented as the mean ± S.D. ∗∗∗*p* < 0.001. *C*, Western blot analysis of Flag-(GR)80 protein levels in HEK293T cells treated with IXA4. The bar graph presents the relative (GR)80 levels post-treatment. Data points are presented as the mean ± S.D. ∗∗∗*p* < 0.001. *D*, Dot blot assay illustrating the effects of ectopic expression of IRE1 and Xbp1s, as well as IXA4 treatment, on endogenous poly(GR) levels in fibroblasts derived from C9orf72-ALS patients. The bar graph displays relative poly(GR) quantification. Data points are presented as the mean ± S.D. ∗∗∗*p* < 0.001.

Given the robust suppression of poly(GR) expression by the IRE1/Xbp1 axis observed in *Drosophila*, we next investigated whether this regulatory mechanism is conserved in mammalian systems. Immunofluorescence staining was performed in HeLa cells transfected with Flag-GR80 alone or co-transfected with Flag-GR80 and either IRE1-GFP or Xbp1s-GFP. Consistent with findings in the fly model, ectopic expression of IRE1 or Xbp1s markedly reduced poly(GR) signal intensity, as visualized by anti-Flag and anti-GFP immunostaining (Fig. 2*A*). Western blot analysis in HEK293T cells further confirmed the reduction of poly(GR) protein levels following IRE1 or Xbp1s overexpression, with EGFP included as a control to indicate comparable transfection efficiency (Fig. 2*B*). To assess whether pharmacological activation of IRE1 affects poly(GR) levels, we treated poly(GR)-expressing cells with IXA4, a selective IRE1 activator (42). IXA4 treatment resulted in a clear reduction of poly(GR) protein levels, further supporting the role of IRE1 in promoting poly(GR) clearance (Fig. 2*C*).

In our previous work, we demonstrated the presence of poly(GR) dipeptide aggregates in the mitochondria of fibroblasts derived from a patient with C9ALS carrying *G4C2* repeats in the *C9ORF72* gene (40). To determine whether activation of the IRE1/Xbp1 axis can mitigate poly(GR) accumulation in patient cells, we performed dot blot analysis in fibroblasts overexpressing IRE1 or Xbp1s. Overexpression of either IRE1 or Xbp1s significantly reduced poly(GR) protein levels in patient fibroblasts. Similarly, treatment of the fibroblasts with 150 μM IXA4 for 24 h led to a pronounced decrease in poly(GR) signal, as confirmed by dot blot analysis (Fig. 2*D*). Collectively, these findings demonstrate that activation of the IRE1/Xbp1 pathway promotes the clearance of poly(GR) in both mammalian cell lines and patient-derived fibroblasts, suggesting a conserved role for this signaling axis in modulating poly(GR) proteostasis and alleviating its associated cellular toxicity in ALS.

### RNA-seq analysis identifies hsp70Ba as a key transcriptional target of the IRE1/Xbp1 axis

**Figure 3:**
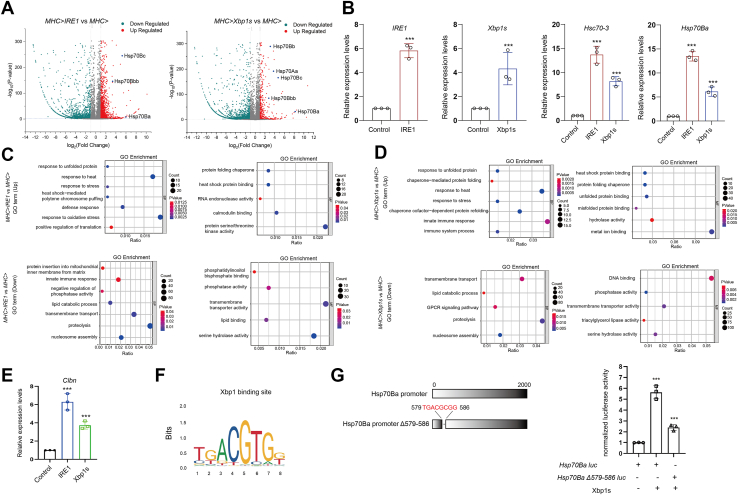
**RNA-Seq analysis reveals Hsp70Ba as a downstream target of the IRE1/Xbp1 pathway.***A*, RNA-seq analysis of IRE1 or Xbp1s overexpression in *Drosophila* muscle, shown with volcano plots highlighting differentially expressed genes. *B*, qRT-PCR analysis quantifying mRNA levels of *IRE1*, *Xbp1s*, *Hsc70 to 3*, and *Hsp70Ba*. Data points are presented as the mean ± S.D. ∗∗∗*p* < 0.001. *C*, gene Ontology (GO) enrichment analysis of genes significantly upregulated or downregulated following IRE1 overexpression. *D*, GO enrichment analysis of genes significantly upregulated or downregulated following Xbp1s overexpression. *E*, Quantitative RT-PCR analysis measuring the mRNA levels of *Clbn*, a key component of the ribosome quality control (RQC) pathway. Data are presented as mean ± S.D. ∗∗∗*p* < 0.001. *F*, predicted Xbp1s binding sites within the *Hsp70Ba* promoter region, as identified using the JASPAR database. *G*, dual luciferase reporter assay demonstrating the effect of Xbp1s on the transcriptional activity of *Hsp70Ba-luc* and the deletion mutant *Hsp70Ba Δ579-586-luc* constructs. Data are presented as mean ± S.D. ∗∗∗*p* < 0.001.

To elucidate the molecular mechanisms by which the IRE1/Xbp1 axis regulates poly(GR) proteostasis, we conducted RNA sequencing (RNA-seq) on *Drosophila* muscle cells. RNA-seq datasets were included in Table S1 (*MHC>IRE1 vs. MHC>*) and Table S2 (*MHC>Xbp1s vs. MHC>*), listing all detected transcripts with corresponding log_2_ fold changes and adjusted *p*-values. Transcriptomic analysis revealed a significant upregulation of multiple members of the Hsp70 family of heat shock proteins upon overexpression of IRE1 and Xbp1s, including *Hsp70Ba, Hsp70Bc, Hsp70Bb* (Fig. 3*A*). Among the IRE1/Xbp1s-induced genes, *Hsp70Ba* exhibited the pronounced transcriptional induction and is known to function as a key cytosolic chaperone involved in maintaining proteostasis. Given its critical role in protein quality control and its strong transcriptional response to IRE1/Xbp1s activation (44, 45), *Hsp70Ba* was selected for further investigation.

Quantitative RT-PCR further validated the transcriptional induction of *Hsp70Ba* and *Hsc70 to 3*, the latter being a previously established target of Xbp1s (33, 46), thereby confirming the regulatory influence of the IRE1/Xbp1s pathway (Fig. 3*B*). Gene Ontology (GO) enrichment analysis identified several biological processes and signaling pathways activated by IRE1 and Xbp1s, including the unfolded protein response and cellular stress response pathways (Fig. 3, *C* and *D*). We also observed that overexpression of IRE1 and Xbp1s resulted in significant transcriptional upregulation of *Clbn*, the *Drosophila* homolog of Nuclear Export Mediator Factor (NEMF), a critical component of the ribosome-associated quality control (RQC) pathway. Quantitative real-time PCR (qRT-PCR) analysis confirmed elevated *Clbn* mRNA levels (Fig. 3*E*). In the RQC pathway, Clbn plays an essential role in surveilling and degrading incomplete proteins arising from defective translation. Specifically, Clbn recruits tRNA-Ala and tRNA-Thr to append C-terminal alanine-threonine (CAT) tails to stalled polypeptides, a process termed CAT-tailing (19, 21, 47, 48). These modified polypeptides are subsequently recognized by the E3 ligase Ltn1 for ubiquitination and targeted for proteasomal degradation, thereby mitigating the accumulation of potentially toxic aberrant proteins.

To elucidate the mechanism by which IRE1 and Xbp1s overexpression upregulates the cytoplasmic chaperone *Hsp70Ba*, the *Hsp70Ba* promoter region was analyzed. Bioinformatic analysis using JASPAR, a database of transcription factor binding profiles, identified a conserved Xbp1s binding site (TGACGCGG) within the 579 to 586 region of the *Hsp70Ba* promoter (Fig. 3*F*). A dual-luciferase reporter assay further confirmed that Xbp1s significantly enhances Hsp70Ba promoter activity, whereas deletion of the predicted binding site partially attenuated this transcriptional activation (Fig. 3*G*). Collectively, these findings indicate that Hsp70 is a transcriptional target of the IRE1/Xbp1s axis and may play a central role in mediating its proteostatic functions.

### Regulation of poly(GR) proteostasis *via* the Hsp70Ba and Rictor/AKT/VCP pathway

**Figure 4:**
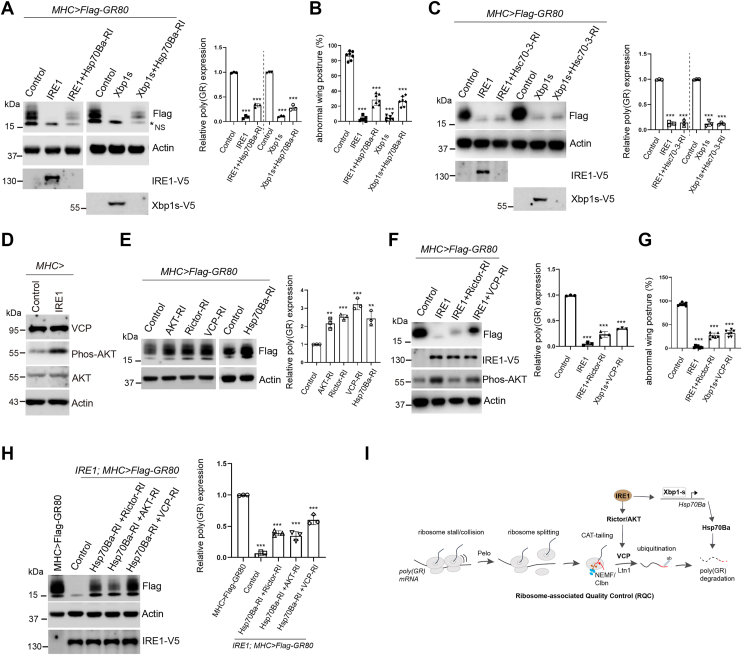
**Regulation of poly(GR) protein levels by the IRE1/Xbp1 axis requires Hsp70Ba and the Rictor/AKT/RQC pathway.***A*, Western blot analysis demonstrating the effect of Hsp70Ba knockdown on the IRE1-or Xbp1s-mediated clearance of Flag-(GR)80 protein in *Drosophila* muscle cells. Quantification of relative (GR)80 protein levels is presented in the accompanying bar graph. Data are presented as the mean ± S.D. ∗∗∗*p* < 0.001. *B*, quantification of the abnormal wing posture phenotype in flies co-expressing Flag-(GR)80 with control, IRE1, IRE1+*Hsp70*Ba-RNAi, Xbp1s, Xbp1s + *Hsp70*Ba-RNAi. Data are presented as the mean ± S.D. ∗∗∗*p* < 0.001. *C*, Western blot analysis showing the effect of Hsc70 to 3 knockdown on IRE1-or Xbp1s-mediated reduction of Flag-(GR)80 protein in *Drosophila* muscle cells, with quantification presented in the accompanying bar graph (mean ± S.D.; ∗∗∗*p* < 0.001). *D*, Western blot analysis showing VCP, AKT, p-AKT levels upon IRE1 overexpression. *E*, Western blot analysis showing the effect of Rictor, AKT, VCP, and Hsp70Ba knockdown on Flag-(GR)80 protein levels, with quantification of relative (GR)80 abundance (mean ± S.D.; ∗∗*p* < 0.01, ∗∗∗*p* < 0.001). *F*, Western blot analysis showing the impact of Rictor or VCP loss on IRE1-mediated clearance of Flag-(GR)80 in *Drosophila* muscle cells. Bar graph shows the quantification of (GR)80 protein levels. Data points are presented as the mean ± S.D. ∗∗∗*p* < 0.001. *G*, quantification of abnormal wing posture in flies co-expressing Flag-(GR)80 with control, IRE1, IRE1+ *Rictor*-RNAi, or IRE1+*VCP*-RNAi. Data points are presented as the mean ± S.D. ∗∗∗*p* < 0.001. *H*, Western blot analysis showing simultaneous inhibition of Rictor/AKT/VCP with HSP70Ba on IRE1-mediated clearance of Flag-(GR)80, with quantification in the accompanying bar graph (mean ± S.D.; ∗∗∗*p* < 0.001). *I*, schematic model illustrating the proposed molecular mechanisms by which IRE1 and Xbp1s regulate poly(GR) protein abundance through Hsp70Ba and the Rictor/AKT/VCP axis.

To determine whether Hsp70Ba contributes to the regulation of poly(GR) protein homeostasis, we performed Hsp70Ba knockdown in *Drosophila* expressing poly(GR) in the genetic backgrounds of IRE1 or Xbp1s overexpression. Loss of Hsp70Ba partially restored poly(GR) protein levels, indicating that Hsp70Ba modulates IRE1/Xbp1s-mediated effects (Fig. 4*A*). Consistently, ectopic expression of IRE1 or Xbp1s alleviated the abnormal wing posture phenotype, whereas inhibition of Hsp70Ba partially restored this phenotype (Fig. 4*B*). We also examined *Hsc70 to 3*, an ER-resident chaperone that facilitates protein folding and regulates IRE1 activation by binding its luminal domain to prevent oligomerization (49). Notably, knockdown of *Hsc70 to 3* in flies expressing *MHC>Flag-GR80* with IRE1 or Xbp1s overexpression did not restore poly(GR) levels (Fig. 4*C*). The efficiency of RNAi-mediated knockdown of IRE1, Xbp1, *Hsp70Ba*, Rictor, AKT, VCP, and *Hsc70 to 3* was confirmed by qRT-PCR (Fig. S2).

We previously demonstrated that the Rictor/AKT/VCP pathway is involved in ribosome-associated quality control of poly(GR). Specifically, poly(GR)-induced ribosome stalling triggers CAT-tailing mediated by Clbn, while the E3 ubiquitin ligase Ltn1 ubiquitinates stalled nascent chains and VCP extracts these aberrant peptides from the ribosome for proteasomal degradation (19). Additionally, Rictor knockdown partially attenuates the TDP-43, FUS, and APP-mediated reduction in poly(GR) expression (20). To investigate whether IRE1 overexpression affects Rictor/AKT/VCP activity, we performed Western blot analyses to assess phospho-AKT, total AKT, and VCP levels in muscle tissue. While total AKT and VCP protein levels remained unchanged, a marked increase in phospho-AKT was observed upon IRE1 overexpression (Fig. 4*D*). Furthermore, inhibition of Rictor, AKT, VCP, or *Hsp70Ba* prevented degradation of poly(GR) (Fig. 4*E*).

To examine whether IRE1 modulates poly(GR) protein abundance through the Rictor/AKT/VCP axis, we knocked down Rictor or VCP in *Drosophila* muscle cells. IRE1 overexpression led to increased levels of phosphorylated AKT, whereas Rictor knockdown suppressed this phosphorylation. Treatment with the IRE1 activator IXA4 also elevated AKT phosphorylation of AKT and expression of endogenous HSP70 proteins (Fig. S3). Inhibition of either Rictor or VCP partially restored poly(GR) protein levels suppressed by IRE1 overexpression, supporting the involvement of the Rictor/AKT/VCP pathway in IRE1-mediated protein quality control (Fig. 4*F*). Similarly, Rictor or VCP interference partially restored the abnormal wing posture phenotype (Fig. 4*G*). Notably, suppression of either the IRE1/Xbp1/*Hsp70Ba* axis or the Rictor/AKT/VCP pathway individually resulted in partial restoration of poly(GR), whereas simultaneous inhibition of both pathways produced a more pronounced rescue, suggesting that these pathways function in parallel to facilitate poly(GR) degradation (Fig. 4*H*).

Previous studies have suggested that artificial activation of the unfolded protein response (UPR) can ameliorate disease features in multiple preclinical models of ALS/FTD (50, 51). Activation of the UPR in response to protein misfolding within the endoplasmic reticulum (ER) initiates protein degradation pathways aimed at restoring ER homeostasis. The UPR induces ER-associated degradation (ERAD) and autophagy, both of which contribute to the clearance of misfolded proteins. To determine whether the IRE1/Xbp1s-mediated RQC pathway preferentially targets poly(GR) proteins for degradation or whether clearance reflects a general enhancement of proteostasis triggered by enforced UPR activation, we performed Western blot analyses of ATP5A, Tom20, Calnexin, and UAS-GFP in flies expressing *MHC>IRE1* or *MHC>Xbp1s*. These proteins were largely unaffected, suggesting that the degradation effect is selective rather than a result of global proteostasis enhancement (Fig. S4).

Taken together, these findings indicate that IRE1 mediates poly(GR) clearance through two distinct mechanisms (1): transcriptional upregulation of Hsp70Ba chaperones, which promotes proper folding and degradation of poly(GR), and (2) activation of the Rictor/AKT/VCP pathway, which resolves translational stalling and facilitates degradation of nascent poly(GR) peptides (Fig. 4*I*). These results highlight the central role of IRE1 in regulating poly(GR) proteostasis and offer potential therapeutic insights for ALS intervention.

### Boosting of IRE1 ameliorate ALS diseases phenotypes in C9orf72 mice

**Figure 5:**
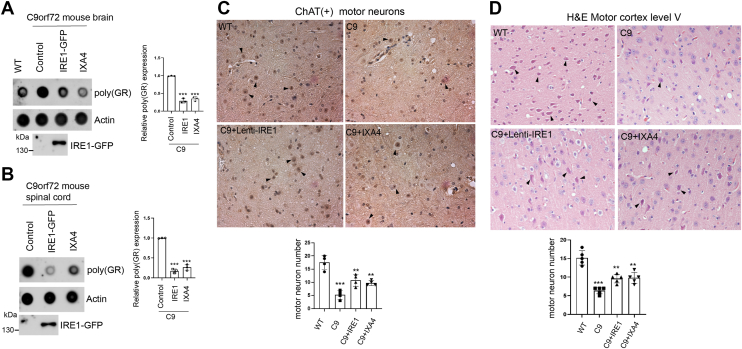
**Activation of IRE1 alleviates motor neuron degeneration in *C9orf72* transgenic mice.***A* and *B*, dot blot analysis showing the effects of ectopic IRE1 expression *via* Lenti-IRE1 or pharmacological activation with IXA4 on poly(GR) levels in the brain and spinal cord of *C9orf72* mice. Quantification of relative poly(GR) protein levels is presented in the accompanying bar graphs. Data points are presented as the mean ± S.D. ∗∗∗*p* < 0.001. *C*, representative immunohistochemistry (IHC) images of motor neurons stained with ChAT antibody following Lenti-IRE1 delivery or IXA4 treatment. Quantification of motor neuron counts per region is shown in the bar graph. Data points are presented as the mean ± S.D. ∗∗*p* < 0.01, ∗∗∗*p* < 0.001. *D*, representative hematoxylin and eosin (H&E) staining images of motor neurons in cortical layer V, following Lenti-IRE1 or IXA4 administration. Quantification of motor neuron numbers per region is displayed in the bar graph. Data points are presented as the mean ± S.D. ∗∗*p* < 0.01, ∗∗∗*p* < 0.001.

To investigate whether IRE1α activation contribute to protects against poly(GR) induced neurodegeneration, we employed the c9ALS/FTD BAC transgenic mice (52, 53). These C9 mice express a human C9orf72 gene with *G4C2* repeats and allow study of progressive disease (54, 55). Firstly, we measured the expression level of the poly(GR) protein in the C9 mice. We found that the expression of poly(GR) protein in the C9 mice was significantly higher than that in normal individuals. We then found that when injecting the IRE1α lentivirus intravenously into 13-month-old mice, it strongly suppressed the expression of poly(GR). Furthermore, after continuous intraperitoneal injection of IXA4 a pharmacological activator of IRE1α in mice for 1 month, the expression level of the poly(GR) protein also significantly decreased in the brain and spinal cord (Fig. 5, *A* and *B*). Pathological analysis of motor neurons revealed loss of ChAT + motor neurons in the lumbar spinal cord and large pyramidal neurons in the motor cortex of C9 mice. After IRE1α lentivirus and IXA4 drug treatment, the morphology of the motor neurons was significantly restored (Fig. 5, *C* and *D*). These results indicate that alteration in IRE1α activation reduces poly(GR) protein levels, alleviates neurotoxicity of C9-ALS/FTD pathology in motor neurons.

## Discussion

*GGGGCC* hexanucleotide repeat expansions in the *C9orf72* gene lead to the production of toxic dipeptide repeat (DPR) proteins *via* repeat-associated non-AUG (RAN) translation. Maintaining poly(GR) protein homeostasis is therefore critical, given that poly(GR) is particularly pathogenic due to its high aggregation propensity and its disruption of essential cellular processes.

Building on our previous findings that translationally stalled poly(GR) is targeted by the ribosome-associated quality control (RQC) pathway, the present study identifies the IRE1/Xbp1s signaling axis as a critical modulator of poly(GR) proteostasis. We demonstrate that overexpression of IRE1 or its downstream effector Xbp1s, as well as pharmacological activation of IRE1 using the small molecule IXA4, significantly reduces poly(GR) protein levels across multiple experimental systems, including *Drosophila* muscle, mammalian cell lines, patient-derived fibroblasts, and a *C9orf72* transgenic mouse model. These findings extend the role of IRE1 beyond its canonical function in the unfolded protein response (UPR), highlighting its broader involvement in proteostasis regulation.

Previous studies have shown that IRE1 contributes to the degradation of misfolded rhodopsin *via* both proteasomal and lysosomal pathways (34). Moreover, emerging evidence has implicated the IRE1/Xbp1s axis in modulating neurodegenerative processes, including amyloid-β accumulation and tau hyperphosphorylation, suggesting a context-dependent role in disease progression (56, 57). Transcriptomic analyses in our study reveal that IRE1/Xbp1s signaling induces the expression of molecular chaperones, particularly members of the Hsp70 family, which facilitate the degradation and clearance of poly(GR). Hsp70 is a key cytoplasmic chaperone essential for maintaining proteostasis and mediating cellular responses to stress. Prior studies have demonstrated that exogenous delivery of Hsp70 delays disease onset, improves motor function, and prolongs survival in ALS mouse models (58). Hsp70 also cooperates with UBQLN2 to mitigate poly(GA)-induced neurotoxicity in ALS/FTD models (59), implying a potentially similar mechanism in counteracting poly(GR) accumulation. In addition, our data implicate the Rictor/AKT/VCP signaling axis-previously linked to translational quality control and RQC-in promoting poly(GR) turnover. Our findings suggest that IRE1/Xbp1s promotes poly(GR) clearance through a dual mechanism: upregulation of Hsp70 chaperones and engagement of the Rictor/AKT/VCP pathway.

Zinc finger protein 598 (ZNF598), a RING-domain E3 ubiquitin ligase, has been implicated as a critical component of the RQC pathway (60). In addition, a separate study demonstrated that ZNF598 plays a role in the co-translational surveillance of poly(GR) proteins (61). While our current study primarily focuses on the IRE1/Xbp1 pathway, it is plausible that ZNF598 acts in coordination with this pathway to promote poly(GR) clearance. Its potential involvement suggests an important and complementary mechanism of co-translational quality control contributing to poly(GR) proteostasis.

Although a recent study reported that inhibition of IRE1 reduced poly(GR)-mediated neurotoxicity in iPSC-derived neurons from C9ALS/FTD patients (62), our findings are consistent with two independent reports demonstrating that enforced expression of Xbp1s attenuated disease progression in models of *C9orf72*-associated ALS/FTD (50, 51). In summary, our results establish the IRE1/Xbp1s axis as a central regulator of poly(GR) degradation and provide strong support for its therapeutic targeting in *C9orf72*-associated ALS and FTD.

## Materials and methods

### *Drosophila* stocks and mice

All *Drosophila* cultures and genetic crosses were conducted using standard procedures at the specified temperatures. Unless otherwise indicated, flies were maintained at 25°C under a 12-h light/dark cycle. Fly food was prepared using a standard formulation consisting of 25 g/L yeast, 66.8 g/L yellow cornmeal, 9.18 g/L soy flour, 40 g/L sucrose, 42.4 g/L maltose, and 6 g/L agar powder. *Drosophila* stocks were obtained from the Bloomington Stock Center and VDRC. MHC>Flag-GR80 (19), IRE1-V5, Xbp1s-V5 (63, 64), IRE1-RNAi (V#39561), Xbp1-RNAi (V#109312), Hsp70Ba-RNAi (BL#43289), Rictor-RNAi (BL#31527), Rictor-RNAi (BL#36699), VCP-RNAi (V#24354), VCP-RNAi (BL#32869), AKT-RNAi (BL#33615), and Hsc70-3-RNAi (BL# 32402). The C9 transgenic mouse line, which expresses the human *C9orf72* gene with *G4C2* repeats, was obtained from Jackson Laboratory. Mice were housed and bred in the Shandong University animal facility under controlled conditions (22 °C, 50% humidity, 12-h light/dark cycle). All animal experiments were approved by the Ethics Committee of Shandong University.

### *Drosophila* behavioral assays

For wing posture analyses, male flies were evaluated at 7 days of age. All experimental groups were aged at 25 °C. For each genotype, 20 male flies were raised in individual vials, and five independent vials were analyzed. Climbing assays were performed by placing 20 flies in an empty culture vial, tapping them to the bottom, and recording the number of flies that climbed 5 cm within 10 s. Three technical replicates were conducted per vial. For survival analysis, newly eclosed flies were distributed into separate vials and maintained at 25 °C. Survival was recorded daily across three independent replicates per genotype.

### Cell lines

HeLa, HEK293T (from ATCC) and *C9orf72*-ALS patient-derived fibroblast cell lines (19) (from Dr Aaron Gitler, Stanford University) were cultured under standard conditions (DMEM supplemented with 10% fetal bovine serum, 5% CO_2_, at 37 °C). Transfections in HeLa and HEK293T cells were performed using Lipofectamine 3000 (catalog #L3000015, Invitrogen) following the manufacturer’s protocol.

### IXA4 treatments

For *Drosophila* experiments, newly eclosed flies were placed in vials containing instant fly food supplemented with 200 μM IXA4 (TargetMol) and maintained for 6 days. Thoracic tissues were then harvested for subsequent analyses. In the mouse model, male *C9orf72* transgenic mice received daily intraperitoneal injections of IXA4 at 50 mg/kg (prepared in 10% DMSO, 40% PEG400, 5% Tween-80, and 45% saline) for 1 month. Following treatment, brain and spinal cord tissues were collected for dot blot and immunofluorescence staining.

### RNA-seq analysis

Total RNA was extracted from the thoracic tissues of *Drosophila melanogaster* using TRIzol reagent (Thermo Fisher Scientific). RNA concentration and purity were assessed with a NanoDrop One spectrophotometer (Thermo Fisher Scientific). Library preparation and sequencing were performed by Majorbio Bio-Pharm Technology Co. Ltd (Shanghai, China). Briefly, 200 ng of total RNA per sample was used for library construction using the TruSeq RNA Sample Preparation Kit (Illumina). Subsequent steps included first- and second-strand cDNA synthesis, PCR amplification, and gel purification. Cluster generation was carried out on the Illumina cBot platform using the TruSeq PE Cluster Kit v3-cBot-HS, and sequencing was conducted on the Illumina MiSeq platform with the TruSeq SBS Kit v3-HS. Sequencing reads were aligned to the Gencode vM19 reference annotation and quantified using featureCounts (v2.0.0). Differential gene expression analysis between treatment and control groups was performed using DESeq2 in R (v4.4.1). Functional enrichment analyses, including Gene Ontology (GO) analysis was conducted using the DAVID Bioinformatics Resource (v2023q4).

### Quantitative real-time PCR (qRT-PCR)

Total RNA was isolated from *Drosophila* muscle cells using TRIzol reagent. First-strand cDNA synthesis was carried out using the HiScript III First Strand cDNA Synthesis Kit (Vazyme) following the manufacturer’s instructions. Quantitative PCR amplification was performed using the 2× M5 HiPer SYBR Premix EsTaq (Mei5bio) on a qRT-PCR system. Primer sequences used in the analysis are listed below.

Hsp70Ba sense: GAGAATACTTTCAACAAGTTAC.

Hsp70Ba antisense: CATTGGCGATAATCTCAACCT.

Hsc70 to 3 sense: TCCCGATGCCGATCCCGAGG.

Hsc70 to 3 antisense: CGCCAGCACCCTGGTACAGC.

IRE1 sense: ATGGAATCCTGTACTCAGGGAA.

IRE1 antisense: CCCTCCTTACCATCCACTGT.

Xbp1 sense: CAACCTTGGATCTGCCGCAGGG.

Xbp1 antisense: CGCTCCAGCGCCTGTTTCCAG.

Clbn sense: GCCGCCTACCATGTTATTTTGG.

Clbn antisense: GTTCCACGGGATATTTCTCGC.

IRE1 sense: ATGGAATCCTGTACTCAGGGAA.

IRE1 antisense: CCCTCCTTACCATCCACTGT.

Xbp1 sense: CTCGAGTTCGGGATACGCAT.

Xbp1 antisense: CCAGGTTAGATGGTCCAGGC.

Rictor sense: GCGGAGCAAATTACAATTACGC.

Rictor antisense: CCTCCAAACAGAGCATCGAGTA.

AKT sense: AAGAGGGGCGAGCACATAAAG.

AKT antisense: GTAACCCATCAGTCTTCCATCG.

VCP sense:AGTCGCGGTGTCCTTTTCTAC.

VCP antisense: GGACCCTTGACTGAGATGAAGTT.

GAPDH sense: TGAGGCGTTTGTGACTTCTG.

GAPDH antisense: GCTTGAGCTGATTTGTGCAT.

### Western blot analysis

Protein lysates were prepared from *Drosophila* muscle tissues or cultured cells using lysis buffer containing 50 mM Tris-HCl (pH 7.4), 150 mM NaCl, 5 mM EDTA, 10% glycerol, 1% Triton X-100, and a protease inhibitor cocktail. Lysates were centrifuged at 10,000*g* for 5 min at 4 °C, and the resulting supernatant was collected for analysis. Proteins were resolved by SDS-PAGE and transferred onto PVDF membranes (0.45 μm pore size). Membranes were blocked with 5% non-fat milk in TBST for 1 h at room temperature and incubated overnight at 4 °C with primary antibodies. After washing, membranes were incubated with the appropriate secondary antibodies for 2 h at room temperature. Protein signals were detected using an enhanced chemiluminescence (ECL) system. The primary antibodies used included: rabbit anti-GFP (1:3000, 50430-2-AP, Proteintech), rabbit anti-Flag (1:2000, F7425, Sigma), mouse anti-actin (1:5000, A2228, Sigma), rabbit anti-AKT (1:500, 9272, Cell Signaling), rabbit anti-phospho-AKT Ser 473 (1:500, 4060, Cell Signaling), rabbit anti-HSP70 (1:500, 4872, Cell Signaling),mouse anti-V5 (1:1000, SV5-Pk1, Invitrogen), rabbit anti-IRE1α (1:1000, NB100–2324, Novus) rabbit anti-P-IRE1α (1:800, NB100–2323, Novus), rabbit anti-P-JNK (1:800, 9255S, Cell Signaling), mouse anti-Cnx99A (1:200, DSHB), mouse anti-ATP5A (1:2000, ab14748, Abcam).

Secondary antibodies included goat anti-mouse IgG-HRP (sc-2005, Santa Cruz Biotechnology) and goat anti-rabbit IgG-HRP (sc-2004, Santa Cruz Biotechnology).

### Dot blot assay

Tissue lysates from mouse brain and human fibroblasts were prepared in 2× SDS lysis buffer and briefly pre-cleared by centrifugation. Protein concentrations were adjusted to 2 μg/μl and mixed with 2× SDS sample buffer. Samples were spotted onto Hybond-C Super NC membranes (GE Healthcare), air-dried, and blocked in 5% non-fat dry milk in PBS. Membranes were incubated overnight at 4 °C with primary antibodies: rabbit anti-poly(GR) (23978-1-AP, Proteintech) and mouse anti-actin (A2228, Sigma), followed by secondary antibody incubation and chemiluminescent detection using the same protocol as standard western blotting.

### Fly muscle staining

For immunohistochemical analysis in adult *Drosophila* indirect flight muscles, 5-day-old male flies raised at 25 °C were used. A minimum of five individuals per genotype were analyzed, and representative images are presented. Dissected muscle tissues were briefly rinsed in 1× PBS and fixed in 4% formaldehyde (in 1 × PBS containing 0.2% Triton X-100) for 30 min at room temperature. Samples were then blocked with 1× PBS containing 5% normal goat serum for 60 min at room temperature, followed by incubation with primary antibodies overnight at 4 °C. The primary antibody used was rabbit anti-Flag (1:1000; Sigma). After three 15-min washes in PBST (PBS+ 0.1% Tween-20) at room temperature, samples were incubated with Alexa Fluor 568-conjugated secondary antibody (1:1000; Molecular Probes) for 2 h at room temperature. Samples were subsequently mounted using SlowFade Gold Antifade Reagent (Invitrogen).

### Immunohistochemistry of cultured cells

HeLa cells were cultured on ethanol-sterilized coverslips. Cells were washed three times with 1× PBS and fixed in 4% formaldehyde (in 1× PBS) for 30 min at room temperature. Fixed cells were washed and permeabilized with 1× PBS containing 0.2% Triton X-100 for 25 min, then blocked in 5% normal goat serum (in PBS) for 1 h at room temperature. Cells were incubated with primary antibodies overnight at 4 °C. Primary antibodies included rabbit anti-Flag (1:1000; Sigma-Aldrich) and mouse anti-GFP (1:1000; Abmart). Secondary antibodies included DAPI, Alexa Fluor 488, and Alexa Fluor 568-conjugated antibodies (1:1000; Molecular Probes).

### Immunohistochemistry in mouse tissues

Mice were euthanized by cervical dislocation, and fresh tissues were collected and fixed in 4% formalin (Sigma) for 24 h, then transferred to 70% ethanol. Tissue processing, paraffin embedding, and sectioning (4 μm) were performed by the Core Facility and Service Platform at Shandong University. Sections were deparaffinized in xylene and rehydrated through graded ethanol series. Antigen retrieval was carried out using citrate buffer (pH 6.0; Sigma) in a steamer for 30 min, followed by cooling to room temperature for 30 to 40 min. Slides were washed in PBS for 10 min and incubated in 3% hydrogen peroxide (in methanol) for 10 min to quench endogenous peroxidase activity, then washed again in PBS. A serum-free blocking solution was applied for 30 min to reduce nonspecific binding. Primary antibody against choline acetyltransferase (ChAT, Millipore) was applied and incubated overnight at 4 °C. Slides were washed in PBS and incubated with Polymer-HRP secondary antibody (Vector Laboratories) for 30 min at room temperature. After additional PBS washes, peroxidase activity was visualized using DAB substrate (Vector Laboratories), and nuclei were counterstained with modified Harris hematoxylin (Sigma-Aldrich). Slides were dehydrated, coverslipped, and imaged using a SPOT microscope with SPOT 5.6 software. For hematoxylin and eosin (H&E) staining, slides were deparaffinized in xylene, rehydrated through ethanol gradients, and stained with hematoxylin (modified Harris, Sigma-Aldrich) for 1 min. After rinsing under running water for 10 min, slides were counterstained with eosin Y (Sigma-Aldrich) for 30 s, rinsed again for 10 min, and coverslipped after rehydration for histological analysis.

### Statistical analysis

Quantification of Western blot and immunofluorescence results was conducted using ImageJ and GraphPad Prism eight software. Statistical analyses were performed using Student’s *t* test or one-way ANOVA, with data presented as mean ± SD. Statistical significance was denoted as *p* < 0.05 (∗), *p* < 0.01 (∗∗), and *p* < 0.001 (∗∗∗).

## Data availability

The authors confirm that all the data described in this study are contained within the article.

## Supporting information

This article contains supporting information.

## Conflict of interest

The authors declare that they do not have any conflicts of interest with the content of this article.
