# Supplementary material for: IRE1/Xbp1 promotes the clearance of poly(GR) dipeptide repeats in amyotrophic lateral sclerosis
Source: J Biol Chem. 2025 Sep 24;301(11):110764. doi: 10.1016/j.jbc.2025.110764 (PMC12590283; doi:10.1016/j.jbc.2025.110764)
Supplement: Supplemental figures [file mmc1.pdf]

Supplementary Figures

Figure S1. Impact of poly(GR) overexpression on IRE1/JNK signaling

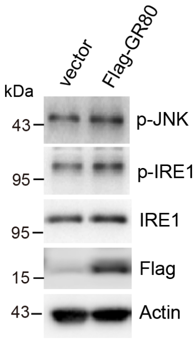

Western blot analysis showing the changes in endogenous IRE1, phosphorylated IRE1 (p-IRE1), and phosphorylated JNK (p-JNK) protein levels in HEK293T cells following overexpression of Flag-GR80.

Figure S2. Validation of RNAi knockdown efficiency

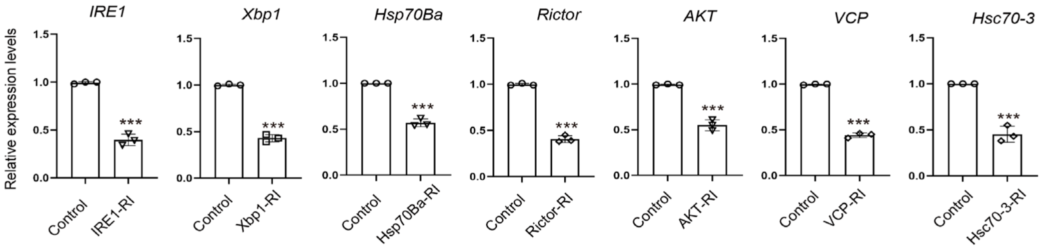

Bar graph demonstrating the knockdown efficiency of *IRE1, Xbp1, Hsp70Ba, Rictor, AKT, and VCP, Hsc70-3*. Data are presented as mean ± S.D. ***P < 0.001.

Figure S3. Effects of IXA4 treatment

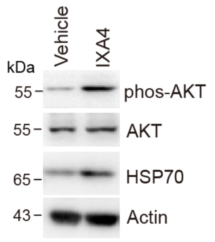

Western blot analysis illustrating the impact of IXA4 treatment on Akt phosphorylation and endogenous HSP70 protein levels in HEK293T cells.

Figure S4. Evaluation of protein abundance upon enforced UPR activation

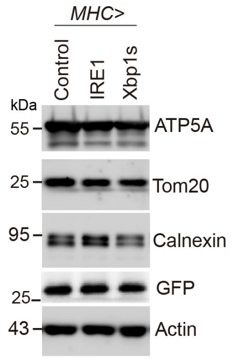

Western blot analysis showing the effects of ectopic expression of IRE1 or Xbp1s on the levels of ATP5A, Tom20, Calnexin, and UAS-GFP in *Drosophila* muscle cells.
