# Supplementary material for: IRE1/Xbp1 promotes the clearance of poly(GR) dipeptide repeats in amyotrophic lateral sclerosis
Source: J Biol Chem. 2025 Sep 24;301(11):110764. doi: 10.1016/j.jbc.2025.110764 (PMC12590283; doi:10.1016/j.jbc.2025.110764)
Supplement: Supplemental Table legends [file mmc2.docx]

**Supplementary table legends**

**Table S1.** **Up and down regulated transcripts upon IRE1 overexpression.** Details of RNA-seq datasets (*MHC>IRE1 vs. MHC>*) with corresponding log_2_ fold changes and adjusted p-values.

**Table S2.** **Up and down regulated transcripts with Xbp1s overexpression.** Details of RNA-seq datasets (*MHC>Xbp1s vs. MHC>*) with corresponding log_2_ fold changes and adjusted p-values.
